# Insights into Genomic Dynamics and Plasticity in the Monkeypox Virus from the 2022 Outbreak

**DOI:** 10.3390/ijms27031371

**Published:** 2026-01-29

**Authors:** Michela Deiana, Elena Locatelli, Laura Veschetti, Simone Malagò, Antonio Mori, Denise Lavezzari, Silvia Accordini, Niccolò Ronzoni, Andrea Angheben, Giovanni Malerba, Evelina Tacconelli, Maria Grazia Cusi, Federico Giovanni Gobbi, Chiara Piubelli, Concetta Castilletti

**Affiliations:** 1 Department of Infectious-Tropical Diseases and Microbiology, IRCCS Sacro Cuore Don Calabria Hospital, Negrar di Valpolicella, 37024 Verona, Italy; elena.locatelli@sacrocuore.it (E.L.); simone.malago@sacrocuore.it (S.M.); antonio.mori@sacrocuore.it (A.M.); denise.lavezzari@sacrocuore.it (D.L.); silvia.accordini@sacrocuore.it (S.A.); niccolo.ronzoni@sacrocuore.it (N.R.); andrea.angheben@sacrocuore.it (A.A.); federico.gobbi@sacrocuore.it (F.G.G.); chiara.piubelli@sacrocuore.it (C.P.); concetta.castilletti@sacrocuore.it (C.C.); 2 Infections and Cystic Fibrosis Unit, Division of Immunology, Transplantation and Infectious Diseases, IRCCS San Raffaele Scientific Institute, 20123 Milano, Italy; laura.veschetti@univr.it; 3 Vita-Salute San Raffaele University, 20123 Milano, Italy; 4 PhD National Programme in One Health Approaches to Infectious Diseases and Life Science Research, Department of Public Health, Experimental and Forensic Medicine, University of Pavia, 27100 Pavia, Italy; 5 GM Lab, Department of Surgical Sciences, Dentistry, Gynaecology and Paediatrics, University of Verona, 37134 Verona, Italy; giovanni.malerba@univr.it; 6 Division of Infectious Diseases, Department of Diagnostic and Public Health, University of Verona, 37129 Verona, Italy; evelina.tacconelli@univr.it; 7 Virology Unit, Department of Medical Biotechnologies, University of Siena, 53100 Siena, Italy; mariagrazia.cusi@unisi.it; 8 Department of Clinical and Experimental Sciences, University of Brescia, 25121 Brescia, Italy

**Keywords:** Mpox, Monkeypox virus (MPXV), 2022 outbreak, short tandem repeats (STRs), viral evolution, whole genome sequencing, Orthopoxvirus, structural modeling

## Abstract

The 2022 global mpox outbreak represented a turning point in the Monkeypox virus (MPXV) epidemiology, highlighting the incredible capability of this virus to adapt to different conditions, also in a non-endemic context. To investigate the genomic dynamics of MPXV 2022 strains, we performed whole-genome sequencing of 40 clinical samples from 16 Italian patients across multiple anatomical sites and timepoints between May and December 2022. Combining single-nucleotide analysis with detailed investigation of short tandem repeats (STRs), we explored inter- and intra-host viral dynamics. We identified 19 STR loci located near or within genes involved in immune modulation and virion morphogenesis. While most STRs remained stable across patients, a subset displayed locus- or matrix-specific variation. Among these, STR-VII—embedded within the coding sequence of OPG153, an envelope-associated protein implicated in viral attachment—showed a 12-nucleotide in-frame deletion, resulting in the loss of four aspartic acid residues (Δ4D variant). Structural modeling indicated that this deletion slightly alters a disordered acidic loop without affecting the global fold, potentially modulating surface charge and immune recognition. Integrating STR profiling into genomic surveillance may enhance resolution in outbreak reconstruction and reveal subtle adaptive processes underlying poxvirus–host interaction and immune escape.

## 1. Introduction

The 2022 global outbreak of mpox caused by the Monkeypox virus (MPXV) marked a turning point in the disease’s epidemiology. Previously considered a rare zoonosis, endemic to Central and West Africa, MPXV expanded rapidly into new geographies and transmission networks.

Over the last few years, MPXV has been described as the most significant species for humans among the Orthopoxvirus genus. Following variola virus (VARV) eradication, concern was raised that MPXV might fill the vacant epidemiological niche previously occupied by VARV [1,2,3]. Since 2017, the incidence of mpox in non-endemic areas had increased, and from May 2022 until August 2025, over 150,000 confirmed cases were reported across more than 137 countries, most of which had no previous history of MPXV transmission [4,5], driven predominantly by human-to-human spread within networks of close or sexual contact [3,6].

The MPXV genome is a double-stranded DNA molecule, featuring two identical oppositely oriented regions of  ~6400 bp in length, known as inverted terminal repeats (ITRs) [6,7]. These genomic regions contain genes assumed to be involved in immune evasion [8,9]; conversely, genes essential for viral replication are located in the highly conserved core region [6,7].

Phylogenetically, MPXV comprises two major clades [10]: clade I (with its subclades Ia and the new Ib, which emerged in 2023 in the South Kivu province), historically restricted to animal populations in Central Africa; and clade II (with its subclades IIa and IIb), restricted to West Africa [11,12]. On 14 August 2024, a public health emergency of international scale was declared due to the increase in mpox cases caused by clade I in the Democratic Republic of Congo and its spread to neighboring countries [5]. Clade Ia was primarily associated with zoonotic transmission from animal reservoirs, but emerging evidence showed that human-to-human transmission has been sustained. In contrast, epidemiological and sequencing information showed that clade Ib MPXV was predominantly associated with human-to-human transmission [5].

The 2022 outbreak was attributed to clade IIb, lineage B.1 [13], and genomic surveillance revealed a surprisingly high number of mutations, including over 40 single-nucleotide substitutions compared to the closest 2018–2019 clade IIb strains. Many of these were APOBEC3-mediated (GA > AA and TC > TT), suggesting ongoing adaptive evolution within human hosts [6].

In the terminal regions of poxviruses’ genomes, gene gain and loss are key drivers of their evolution and adaptation to the hosts [14,15]. Studies have shown that deletions were linked to increased human-to-human transmission [16], and gene copy number variation has been identified as a factor able to modulate viral fitness [17]. Moreover, characterized MPXV genomes from the 2022 outbreak have chimeric sequence architectures (mosaicisms), copy-number variation in tandem repeats, and significant linkage disequilibrium among SNPs, findings that strongly suggest that natural recombination acts alongside mutation and selection in shaping MPXV genomic diversity and its capacity for adaptation to new hosts [18].

Viral genomes often contain low-complexity regions (LCRs), defined as stretches of biased or repetitive sequence with limited nucleotide diversity. These regions frequently harbor short tandem repeats (STRs), i.e., short nucleotide motifs repeated in direct succession, whose copy number can vary among genomes. STRs have been described across a broad range of viruses [19,20,21,22,23], where they have been linked to genome plasticity, regulation of gene expression, host adaptation, and even differences in virulence or immune evasion. Despite growing evidence of tandem-repeat variability, STRs have not yet been fully characterized in MPXV. STR enrichment in low-complexity domains of MPXV suggests they could likewise contribute to viral diversity via mechanisms such as modulation of expression, structural genome plasticity, or genome length variation. A recent study showed that STRs are widespread and highly variable across strains, notably concentrated in the ITRs, with copy-number differences even among closely related isolates [6]. Such features suggest STRs may function as evolutionary “tuning elements,” fostering phenotypic variability in surface proteins and immune modulators with potential consequences for viral fitness, transmission, and immune escape [6].

In this study, we investigated the genomic dynamics of single-nucleotide polymorphisms (SNPs), STRs, and low-complexity regions in MPXV genomes collected during the 2022 outbreak in Italy. We applied a longitudinal design, analyzing clinical specimens collected at multiple timepoints, from diagnosis through follow-up, across different biological matrices.

## 2. Results

### 2.1. QC of Sequencing Data, Read Alignment, and Hybrid Assembly

Sequencing yielded an average of 2’029’939 Illumina and 273’023 ONT reads. Illumina sequencing generated between 101,826 and 9,409,984 total reads per sample, while ONT sequencing generated between 942 and 1’398’497 total reads per sample. Sequencing metrics, QC, and coverage against the RefSeq NC_063383.1 are reported in Supplementary Materials, Table S1. To better recapitulate the number of repeated sequences and to increase the accuracy of the STR analysis, hybrid genome assemblies were built for each sample by combining short and long reads. The hybrid assemblies generated on average 4 contigs belonging to MPXV, covering 97% of the NC_063383.1 reference sequence.

#### 2.1.1. Single Nucleotide Polymorphisms

**Figure 1:**
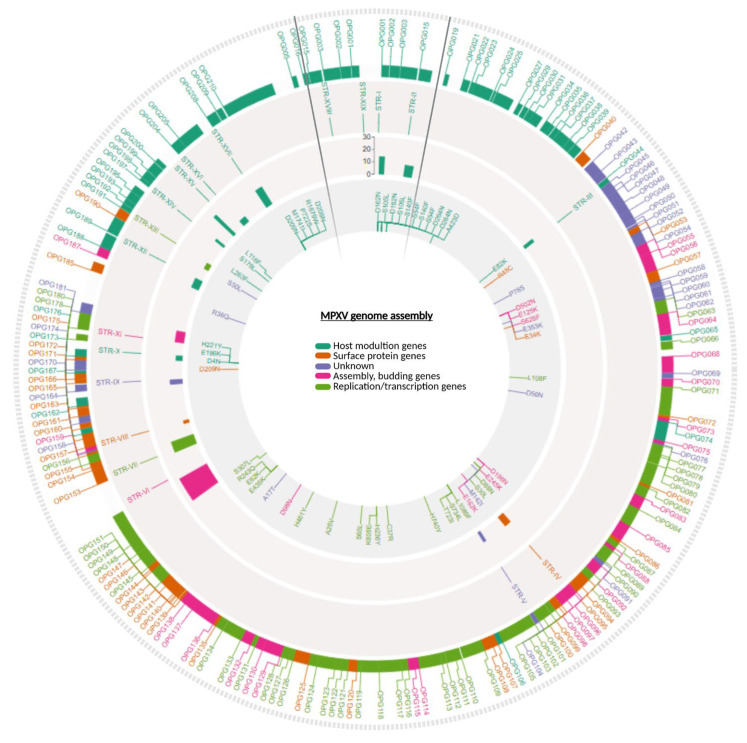
Genomic organization of the MPXV genome, highlighting the distribution of SNPs and STRs across functional gene categories. A visual representation of the fully annotated MPXV assembly genomes (based on the NC_063383 genome annotation). Starting from the outer ring, MPXV genes are displayed and color-coded according to their predicted function: host modulation genes (dark green), surface protein genes (orange), assembly/budding genes (pink), replication/transcription genes (light green), and genes of unknown function (purple). Moving inward, the next track marks the positions of STRs, located either within genes (intragenic) or nearby (upstream or downstream). The following ring shows the number of repeat units for each STR (see scale bar). The innermost circle indicates the annotated SNPs across the genome. The double black lines delimit the ITRs of the MPXV genome.

To explore patterns of viral mutation across samples, we conducted two complementary analyses: (i) exploration of novel nucleotide substitutions across patients, aimed at identifying potential convergences; and (ii) an intra-patient comparison of distinct biological matrices, to assess intra-host variability. Notably, P6, P10, P11, and P12 shared a pattern of five novel mutations (C101983T, G28334A, C162816T, C170573T, and C72215T), suggesting close phylogenetic relatedness since all samples belong to the B.1.7 lineage. Similarly, patients P3 and P4 shared three unique substitutions (G37152A, C184829T, and C152445T). We observed intra-host variability in the number of novel SNPs across different sample types, despite all matrices belonging to the same individual and timepoint. In P3, the skin lesion showed a higher number of novel mutations, followed by the pharyngeal swab and the saliva, which exhibited a reduced SNP profile. Specifically, mutations such as G37152A were consistently detected across all matrices, with the genital lesion carrying the G150394A mutation univocally in the pharyngeal sample. Similarly, in P4, while most matrices carried the full set of novel SNPs, the abdominal skin lesion additionally carried C1092T, G171341T, and G171341T. Figure 1 shows the localization of the identified SNPs along the MPXV genome. Detailed results are reported in Supplementary Materials, Table S2.

#### 2.1.2. Characterization of STRs in MPXV Genomes

We systematically analyzed STRs using Tandem Repeat Finder. A total of 19 STRs (STR-I to STR-XIX) were identified (Table 1). The 19 STR regions were detected across all samples, and varied in length from 9 bp to 144 bp, with repeat units ranging from mononucleotide stretches (e.g., STR-I, STR-III, STR-IV, STR-V, STR-VI, STR-XI, and STR-XIX) to more composite motifs, such as STR-IX and STR-X. The number of repeats spanned from 3.7 (STR-XIII) to 25 (STR-VI), with different levels of conservation across samples. Interestingly, STR-I and STR-XIX, as well as STR-II and STR-XVIII, were located in the ITRs, where they were present as identical copies in reverse-complementary form. Nine STRs were located in proximity to annotated MPXV genes (OPGs), either upstream, downstream, or within ORFs. Most intragenic STRs (e.g., STR-III, -IV, -V, -VIII, -XIII, -XIV, and -XVI) were highly conserved across patients and timepoints.

**Figure 2:**
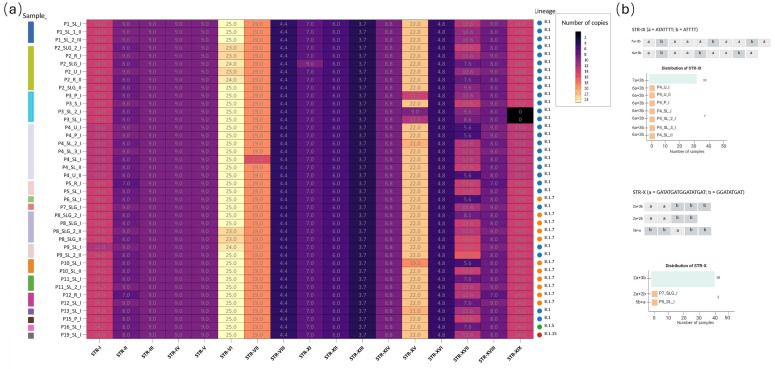
Comprehensive overview of STR copy number and structural variability across all samples. (**a**) Heatmap showing the distribution and the repeat number of the simple repeat motifs. The y-axis lists the samples, identified by sample id, while the x-axis shows the STRs. Color intensity reflects the STR copy number, as indicated by the scale bar. On the right, the lineage of the sequences in analysis is presented. (**b**) STR loci displaying rearrangements in repeat architecture (e.g., STR-IX and STR-X) are illustrated together with the distribution of the observed configurations.

By contrast, STR-X, although stable in copy number, displayed changes in repeat architecture (e.g., 2a + 3b to 2b + a + 3b configurations), highlighting a high structural plasticity at these loci. Non-intragenic loci, such as STR-II, STR-XV, STR-XVII, and STR-XIX, were the most variable, showing larger fluctuations in repeat copy number. The identified STRs and their corresponding repeat numbers are displayed in Figure 1. A comprehensive overview of the STR loci is provided in Figure 2; the loci displaying simple repeat motifs are reported in Figure 2a, while the loci in which we detected rearrangements in the architecture are presented in Figure 2b.

##### Intra-Host STRs Variation Across Matrices and Timepoints

We investigated the distribution of the 19 identified STRs across the 40 sequenced matrices. Overall, STR profiles were highly conserved across patients and sample types. A large number of STR loci, including STR-III, STR-IV, STR-V, STR-VIII, STR-XII, STR-XIII, STR-XIV, and STR-XVI, were unaltered across all samples, and STR-VI showed only minor fluctuations (23–25 repeats) in a subset of matrices. Some other STR loci presented a more variable pattern across samples. For instance, P3 exhibited copy-number variation without locus loss, with a marked reduction in STR-XV in skin lesions (14, 12, and 9 repeats vs. the prevalent 22) and a slight decrease in STR-II in the saliva (8 vs. 9 in other matrices). Notably, STR-XIX was absent in the two skin lesion samples (P3_SL_2_I and P3_SL_I), whereas it was present in saliva and pharyngeal swab.

P4 retained all STR loci across matrices, but consistently displayed lower repeat counts at specific sites compared with the predominant pattern in other patients: STR-I = 13 (vs. 14), STR-IX = 9 (vs. 10) in all P4 matrices, and STR-XIX = 13 (vs. 14). Additional matrix-specific differences included STR-VII = 15 in P4_SL_I (vs. 19 elsewhere) and STR-XV = 21 in P4_SL_2_I (vs. 22 in the other P4 samples).

With the exception of P3 and P4, only sporadic changes were observed: P7_SLG_I showed STR-X = 4 (vs. 5 in all other samples), P10_SL_I had STR-XV = 19, P4_SL_2_I and P13_SL_I had STR-XV = 21, P2_SLG_I showed STR-XI = 9 (vs. 7 elsewhere), and P5_R_I had STR-II = 7. Several loci also exhibited modest dispersion across patients/timepoints, notably STR-XVII (range 5.6–16) and STR-XVIII (range 7–9).

For the composite STRs (i.e., STR-IX and STR-X), beyond the number of repeated sequences changes, we notice that they segregated into two different block configurations. STR-IX is composed by [ATATTTT]n + [ATTTT]n; we defined the block [ATATTTT] as ‘block a, [a]’ and [ATTTT] as ‘block b, [b]’. The majority of analyzed samples accounted for a total of 10 repeats, composed as 7[a] + 3[b]. In all P4 matrices, we identified a total of 9 repeats, coherently with alternative architectures, but with the loss of one block a, resulting in 6[a] + 3[b] (Figure 2b).

Meanwhile, STR-X, defined as [GATATGATGGATATGAT]n (block a) + [GGATATGAT]n (block b), at a first glance, seemed stably conserved across samples, except in one case, which lost one repeat (P7_SLG_I). In 38 out of 40 analyzed samples, the architecture of the STR was 2[a] + 3[b], with a 2[a] + 2[b] variant in P7_SLG_I, resulting in a total of 4 repeats. Interestingly, in P5_SL_I we noticed a motif rearrangement, leading to 2[b]+ [a] +3[b] (Figure 2b). Beyond STR-IX and STR-X, sporadic variability was observed in STR-XV, STR-XVII, and STR-XIX, suggesting that most STR changes are patient- or matrix-specific rather than lineage-associated. Across longitudinal samples from the same patient, STR differences were observed in a limited number of loci, while the majority of STRs showed stable repeat copy numbers.

##### STRs and In Silico Protein Implications

In total, 9 out of the 19 STRs identified were located within annotated genes or partially overlapping the corresponding coding sequences; STR-X and -XIII were positioned in intergenic regions, outside the coding sequence. Analyzing the potential effects of STR variation on the coding sequence, no changes were observed in STR-III, -IV, -V, -XIV, and -XVI when compared with the annotated protein (Supplementary Materials, Figure S1). STR-X represents one of the most intriguing regions. Although located immediately upstream of the OPG176 coding sequence, its repeat block composition (e.g., 2a + 3b vs. 2b + a + 3b) showed rearrangements across samples (P5_SL_I and P7_SLG_I), despite overall conservation in repeat number. This structural variability may not directly alter the encoded protein sequence but could impact transcriptional or translational regulation. In contrast, STR-XIII, located within OPG190 (encoding the secreted interferon α/β decoy receptor B19R), remained fully conserved across all samples, consistent with strong functional constraints on this essential immunomodulatory gene [8].

**Figure 3:**
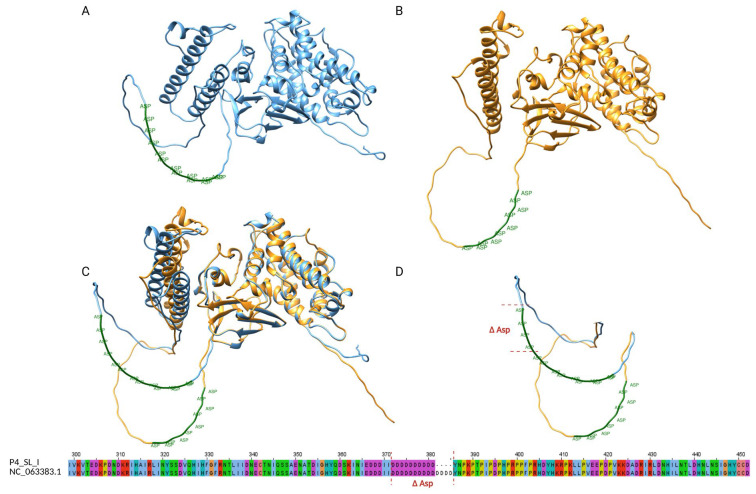
Structural comparison of OPG153 wild-type and Δ4D variant. (**A**) Predicted three-dimensional model of wild-type OPG153 (blue) showing the poly-aspartic region (STR-VII, green). (**B**) Model of the Δ4D variant (yellow), in which the poly-Asp tract is shortened due to the deletion of four aspartic acid residues. (**C**) Overlay of wild-type and Δ4D models highlighting the contraction of the STR-VII acidic loop. (**D**) Close-up view of the poly-Asp region, with the deleted residues indicated in red (ΔAsp). The deletion does not disrupt the overall fold but shortens the acidic loop and slightly alters local charge distribution and flexibility.

Conversely, the skin lesion sample from P4 at timepoint I (P4_SL_I) showed a lower copy number of STR-VII, which is located within the coding sequence of OPG153. Specifically, we identified a 12-nucleotide in-frame deletion that resulted in the loss of four aspartic acid residues in a poly-Asp tract. The OPG153 gene (GeneID: 72551547) spans 1530 bp and encodes for the envelope protein OPG153 (Uniprot ID: A0A7H0DNC4), a surface-associated viral factor implicated in attachment and fusion suppression [9,25,26]. The STR-VII motif corresponds to a low-complexity, negatively charged region described in UniProt as a compositional bias domain. To evaluate potential structural consequences of the Δ4D deletion, both the wild-type and mutant protein sequences were modeled ab initio using AlphaFold2. Structural alignment of the two models revealed a high overlap, with the deletion confined to a disordered loop within the poly-Asp tract (Figure 3). The shortened acidic loop of the Δ4D variant displayed a slightly more compact conformation, while the global fold remained unchanged. These observations suggest that the deletion likely alters local electrostatic properties and loop flexibility.

#### 2.1.3. Phylogenetic Analysis and STR-Based Clustering

**Figure 4:**
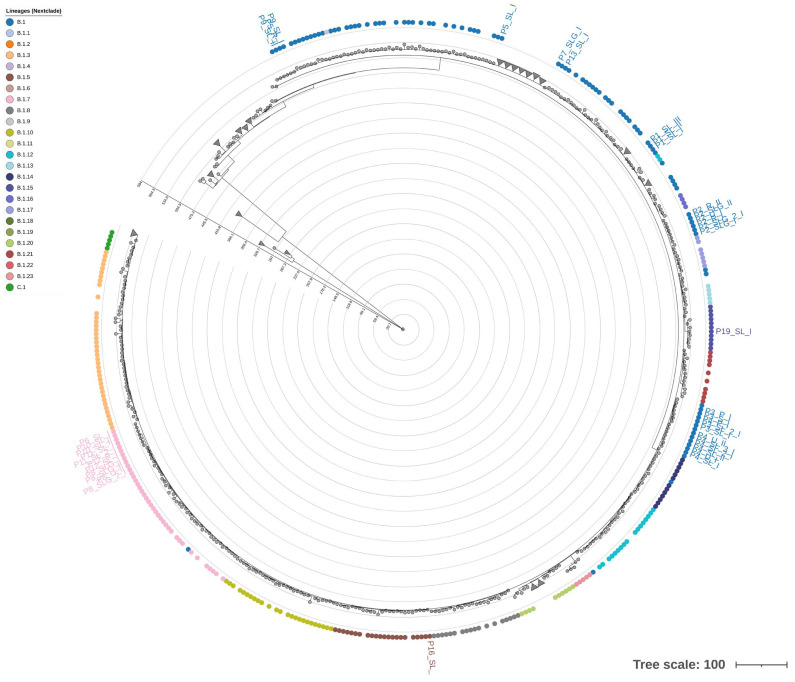
Phylogenetic tree. Circle tips represent samples, colored by lineage (B.1–C.1). Branch lengths reflect genetic distance, shown by the concentric rings; scale bar 100 substitutions. The circles represent the samples used for the tree construction; the triangles represent the collapsed branches. Labels highlight the samples analyzed in this study.

**Figure 5:**
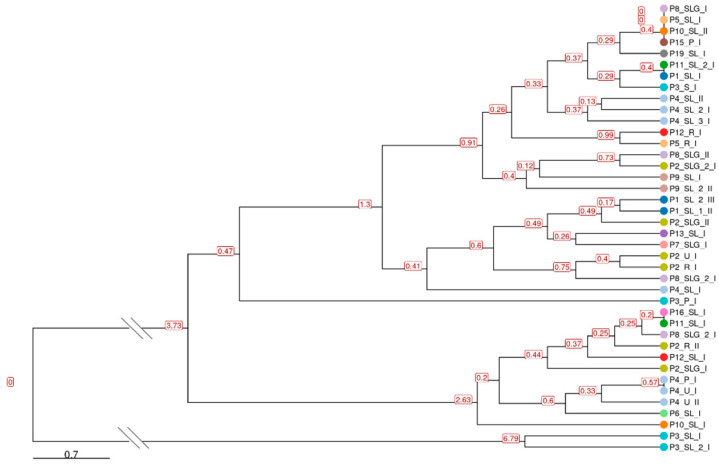
**Hierarchical clustering of STR profiles**. The dendrogram is based on Euclidean distances, which reflect the dissimilarity between STR repeat copy numbers. Shorter distances indicate higher similarity in STR patterns between samples, while longer distances reflect greater divergence. The clustering is derived from repeat copy numbers and does not represent nucleotide sequence relationships.

Phylogenetic reconstruction based on whole-genome sequences clustered all Italian samples within clade IIb, in agreement with global surveillance data from the 2022–2023 outbreak. Of the total 40 sequences, 27 clustered into lineage B.1, 11 clustered into sublineage B.1.7, 1 into B.1.5, and 1 into B.1.15 (Figure 4. The tree revealed short branch lengths, indicating limited nucleotide divergence among circulating strains. Minor intra-lineage clustering patterns were detected, notably among P6, P10, P11, and P12, all belonging to sublineage B.1.7, consistent with their shared SNP profiles. P3 and P4 formed a divergent branch supported by private substitutions, in line with the SNP-based variant analysis. To complement the SNP-based phylogeny, we constructed a hierarchical clustering using the STR copy number matrix (Figure 5). The STR dendrogram showed an overall structure consistent with the SNP-based topology, supporting a high degree of genomic conservation across patients. However, subtle rearrangements in clustering order were observed. For instance, P3 and P4, which cluster together in the ML phylogeny, appeared more distant in the STR-based dendrogram due to variation in STR-VII (OPG153), STR-IX, and STR-X, while P6, P10, P11, and P12 remained closely grouped, mirroring their shared STR configuration. Notably, the dendrogram did not reveal segregation by matrix type or sampling timepoint. Overall, STR-based clustering partially recapitulates SNP-based phylogeny but provides additional resolution at the intra-lineage level. The discordance between the two approaches suggests that tandem-repeat variation evolves independently of point mutations, potentially reflecting rapid, reversible changes driven by polymerase slippage or recombination. These findings support the hypothesis that STRs can act as fast-evolving molecular markers, complementing SNP analyses for high-resolution tracking of MPXV genetic diversity.

## 3. Discussion

In this study, we provide a comprehensive view of the genomic plasticity of MPXV in a cohort of 16 Italian patients during the 2022 outbreak, by integrating SNP-based and STR analyses, and evaluating intra-host variability across timepoints and different anatomical matrices. Our work combines hybrid assemblies reconstruction, with repeat profiling and mutational analysis, revealing interesting features potentially involved in MPXV evolution and adaptation.

The SNP-based phylogenetic reconstruction confirmed the co-circulation of multiple B.1-derived sublineages, including B.1.7, B.1.5, and B.1.15, consistent with broader genomic surveillance [6,27]. However, our novel mutation analysis unveiled unexpected convergences between distinct patients. For instance, five novel mutations (C101983T, G28334A, C162816T, C170573T, and C72215T) were shared among four unrelated patients (P10, P11, P12, and P6), all belonging to lineage B.1.7. Similarly, the shared substitutions between P3 and P4 (G37152A, C184829T, and C152445T) could strengthen the hypothesis of co-circulating, under-sampled variants [28]. Functional annotation of these novel variants indicated that several mapped into or near genes potentially involved in viral fitness or host interaction. For example, OPG002 encodes CrmB, a TNF-binding protein that inhibits inflammatory responses [29]; OPG105 contains ankyrin repeats often associated with inhibition of NF-κB signaling [8]. Some of these SNPs are not yet reported in GISAID or GenBank lineage-defining mutation databases [30], suggesting ongoing intra-host diversification. These subtle differences highlight the importance of sampling multiple anatomical sites to capture the full scope of intra-host viral diversity [28] and capture long-term evolutionary processes, providing a stable phylogenetic reference. While SNPs provide a stable framework for lineage assignment, they do not fully capture the intra-host variability.

Importantly, STR profiling revealed a set of 19 conserved STRs (STR-I to STR-XIX), with high stability across most patients and matrices. STRs in genes involved in host immune interactions (e.g., OPG015, OPG204) showed minimal variability, supporting their potential function as key structural regulators [31].

When stratifying the STR profiles by timepoint (I, II, III), no systematic temporal variability was observed. The distribution of repeated sequences copy numbers remained stable across all timepoints, with the few observed differences attributable to inter-sample rather than longitudinal variation. These alterations did not correlate with coverage issues and were independent of lineage, pointing to intra-host viral dynamics. Our results indicate that intragenic STRs are overall under evolutionary constraint, remaining stable in repeat copy number. It should be acknowledged that, as not all anatomical matrices and timepoints were available for each patient, the heterogeneous longitudinal sampling may have potentially limited the detection of subtle temporal trends.

Nevertheless, STR-IX and STR-X represent notable exceptions, as they exhibit structural reorganization of repeat motifs despite numerical stability. In contrast, variability is concentrated in non-intragenic loci (e.g., STR-XV, STR-XVII, and STR-XIX), which may accumulate changes more freely and contribute to inter-patient differences, in accordance with previous findings on repeat instability at specific MPXV genome sites [32]. These results support the view that STRs may act as “evolutionary tuning knobs,” modulating viral gene expression or protein structure in response to host pressures [16,32]. The association of STRs with genes, such as OPG001 (a chemokine-binding protein) [32], OPG044 (a Bcl-2–like immune modulator) [33,34], OPG104 (an entry/fusion protein) [25,35], and OPG180 (a replication-associated protein subject to APOBEC3-like pressure) [36], suggests that STR instability may intersect with functionally important loci, potentially influencing immune evasion, viral entry efficiency, and replication fidelity. The conservation of most intragenic STRs likely reflects strong functional constraints, as repeat variation within coding regions may impact protein length, domain organization, or local folding. In this context, the architectural changes we observed in STR-IX and STR-X are of particular interest, since they may influence local protein conformation despite numerical stability in repeat copy number.

A particularly interesting case was represented by STR-VII, located within the coding region of OPG153. The skin lesion sample from P4 at timepoint I (P4_SL_I) showed a 12-nucleotide in-frame deletion corresponding to the loss of four aspartic acid residues (Δ4D) in a polyaspartic acid tract. The affected motif is located in a low-complexity, acidic region annotated in UniProt as a compositional bias domain. AlphaFold2-based structural modeling revealed that both wild-type and Δ4D proteins share a highly similar global fold, with the deletion confined to a flexible loop within the poly-Asp region (Figure 3), exhibiting a shorter and more compact loop. Though the functional implications cannot be assessed in this study, these results provide a framework for future investigations aimed at linking repeat architecture to protein structure and viral fitness. A recent work [37] identified broadly neutralizing antibodies targeting OPG153, highlighting its central role in poxvirus immune recognition. While our analyses did not reveal SNP-based variability in this gene, the structural variation observed in its STR region may influence antigenicity. These findings, although derived from in silico modeling and remain hypothesis-generating, further emphasize the importance of the integration of structural and immunological perspectives in future investigations.

The loci, OPG176 and OPG190, exemplify opposite selective regimes acting on STRs. For OPG176, STR-X was located immediately upstream of the coding sequence and maintained a stable overall repeat number, but displayed block-level rearrangements (e.g., 2a + 3b vs. 2b + a + 3b). This architecture plasticity within a non-coding context is consistent with a potential regulatory role, possibly modulating mRNA secondary structure, ribosome accessibility, or translation initiation efficiency (Figure 1). Given that OPG176 encodes a putative component of the entry/fusion complex involved in membrane fusion and virion morphogenesis, even subtle alterations in its expression timing or abundance could influence virion assembly or cell tropism. [25,26] The conservation of overall repeat length despite changes in motif organization suggests potential effects operating at the transcriptional level.

In contrast, STR-XVI, located within OPG190 (encoding the secreted interferon α/β decoy receptor B19R) [8], remained completely conserved across all samples. Maintaining the structural and quantitative integrity of B19R is likely critical for effective antagonism of host interferon responses, leaving little room for repeat drift or rearrangement. OPG176 and OPG190 represent two extremes of STR functional behavior: one permissive and regulatory, the other highly constrained and structurally essential. Taken together, our results suggest that intra-host MPXV genomic plasticity is present in a limited number of loci.

In the context of MPXV genome analysis, it is important to consider the intrinsic complexity of MPXV infections during the 2022 outbreak, during which the presence of different viral subpopulations has been increasingly reported [25,26]. These observations suggest that a single infection may not always be represented by a unique and uniform viral population, particularly when multiple samples are collected from the same individual over time or across different anatomical sites. Within this framework, the differences observed across longitudinal and multi-site samples in our study can be interpreted as part of a broader landscape of genomic variability, which may influence the stability and interpretability of the observed data.

Together, these findings suggest the interplay between SNP mutations and STR variability in shaping MPXV genomic structure and driving its evolution. While SNPs reflect cumulative mutation processes and lineage divergence, STRs appear more sensitive to intra-host forces, capturing short-term dynamics and/or anatomical compartmentalization. Most STR loci and SNP profiles were indistinguishable among patients sampled during the summer period (May–August), consistent with close epidemiological linkage. In contrast, patients sampled later in the year (P3, P4, September–December) carried additional private SNPs and showed slightly more heterogeneity at selected STR loci. This pattern may reflect temporal divergence or independent introductions, although further analyses would be required to confirm this hypothesis. The perspective of a combination of SNP and STR variability reveals that, even in a DNA virus with relatively low mutation rates, MPXV could exhibit notable genomic flexibility [38].

Monitoring both SNPs and STRs may improve molecular epidemiology efforts from a public health standpoint. STR configurations could be key complementary markers for phenotypic traits definition, such as immune escape. The current results show, for the first time, the presence of matrix- and patient-dependent SNP and STR patterns within single individuals, supporting a scenario of intra-host viral microevolution. Such a phenomenon, while typically associated with chronic RNA virus infections, may also occur in Orthopoxvirus infections and contribute to phenotypic or transmission potential variability [6,39,40].

In conclusion, our longitudinal, multi-matrix study offers an interesting glimpse into the intra-host evolution of MPXV. However, late autumn cases reveal that the viral genome can undergo localized changes, potentially influenced by tissue-specific pressures or immune compartmentalization. These include not only reductions in repeat copy number but also alternative motif architectures in composite STRs (e.g., STR-IX and STR-X). These dynamic features need further investigation in larger cohorts and may have implications for transmission fitness and pathogenesis. These findings pave the way for future studies aimed at integrating genomic observations, such as matrix-specific STR patterns and novel SNP profiles, with detailed clinical data, including disease severity, symptom localization, and immune response. This integrative approach may help clarify whether intra-host genomic variability correlates with phenotypic manifestations and tissue tropism, ultimately improving our understanding of MPXV pathogenesis and transmission dynamics.

## 4. Materials and Methods

Clinical specimens were collected from patients presenting with skin lesions and clinical manifestations suggestive of mpox infection. Samples were initially submitted for diagnostic purposes to the regional referral laboratory at IRCCS Sacro Cuore Don Calabria Hospital, Negrar di Valpolicella (Verona, Veneto Region, Italy).

A total of 16 patients were confirmed to have mpox by qPCR (Monkeypox Virus Real-Time PCR Kit, BioPerfectus, Taizhou City, China), with cycle threshold (Ct) values ≤ 28. Samples yielding these Ct values were selected for full-genome sequencing.

Following diagnosis, all 16 confirmed patients were enrolled in a longitudinal follow-up protocol. Weekly samples were collected from multiple anatomical sites, including skin lesions, saliva, urine, and pharyngeal swabs, starting from the time of diagnosis and continuing until viral clearance, defined by two consecutive negative qPCR results. A total of 4 patients completed two follow-up visits, and 1 patient completed three visits.

In total, 40 clinical samples from 16 patients were included in the sequencing analysis. All patients were male and received a diagnosis of mpox between May and November2022. Patients’ demographic characteristics and the types of collected specimens are summarized in Table 2.

All samples analyzed in this study were derived from routine clinical diagnostics and were retrospectively included. Although additional follow-up specimens were available for some patients, only samples meeting predefined qPCR Ct thresholds were suitable for whole-genome sequencing and were therefore included. As a consequence, the number of longitudinal and multi-site samples varied across patients.

### 4.1. DNA Extraction, Library Preparation, and Sequencing

Viral DNA was extracted from 200 µL of each matrix and eluted in 60 µL using an EZ1 Advanced XL instrument and EZ1 DSP Virus Kit (Qiagen, Hilden, Germany) according to the manufacturer’s instructions. DNA concentration was determined using the Qubit dsDNA HS assay kit (Thermofisher, Waltham, MA, USA). For short-read sequencing, a target enrichment based on hybrid capture was carried out using the Illumina RNA prep with an enrichment kit and the VSP panel v1 (Illumina, San Diego, CA, USA). The quality of libraries was assessed using the genomic ScreenTape kit and the 4200 TapeStation System (Agilent, Santa Clara, CA, USA). Illumina libraries were prepared according to the manufacturer’s instructions and run on a NextSeq1000 using the P1 300-cycle Illumina flow cell to perform 2 × 150 paired-end sequencing. For long-read Oxford Nanopore Technology (ONT) sequencing, enrichment was performed by generating 2,5 kbp-amplicons24. Shortly, two separate reactions were set up per sample as follows: 6.88 μL nuclease-free water, 0.625 μL primers pool 1 or 2 (100 μM), 12.5 μL Q5 High-Fidelity 2X master mix (New England Biolabs, Ipswich, MA, USA), and 5 μL of sample DNA. The amplification reaction was set up with the following parameters: 98 °C for 1 min, then 35 cycles of 98 °C for 10 s, 65 °C for 30 s, 69 °C for 100 s, and a final extension at 72 °C for 2 min. Separate reactions were then pooled for each sample, and 200 fmol of DNA was used to prepare sequencing libraries with the Native Barcoding Sequencing Kit (SQK-NBD114.24 Oxford Nanopore Technologies, Oxford, UK) as per the manufacturer’s instructions. ONT libraries were run on a MinION Mk1d instrument using FLO-MIN114 (Oxford Nanopore Technologies, Oxford, UK) flow cells to a target of 100× coverage per sample.

### 4.2. Bioinformatic Analysis

Read QC was performed with fastp v0.23.4 and fastqc v0.12.1. Human reads were removed using Kraken2 v2.1.3 [41]. Reads were aligned to the MPXV reference genome (NC_063383.1) using BWA-MEM v2.2.1 [42]; variants were called with bcftools v1.20 [43], filtering for Q > 30, mapQ > 50, DP > 20.

#### 4.2.1. Hybrid Assembly

In order to explore the STR variability, hybrid assemblies were generated using Unicycler v0.5.1 [44], combining Illumina and ONT reads, oriented with MUMmer/Nucmer v4.0.1 [45], and finally, the oriented contigs were assembled into a single scaffold using a homemade script. This script concatenates the aligned contigs while accounting for gaps within and between them, allowing for their accurate identification and distinction. The correspondence between the experimental ID (reported in this manuscript) and the GISAID IDs (EPI_IDs) is provided in Supplementary Materials, Table S3.

#### 4.2.2. STR Analysis

Short tandem repeats were identified using Tandem Repeat Finder v.4.09 [46] (parameters “2 7 7 80 10 50 2000 -ngs”), retaining loci with entropy > 1.8, match ≥ 80%, and non-overlapping regions. Partial repeats were also considered. Tandem Repeat Finder detects repeated patterns without requiring prior specification, but it does not detect single-nucleotide repeats (i.e., homopolymers). A custom R script using a cut-off of at least 9 nucleotides was systematically identified to detect the homopolymer repeats. Then, the identified STRs were visually inspected in the raw reads.

All loci were manually curated.

#### 4.2.3. Global Dataset, Phylogeny, and STR-Based Clustering Analysis

Complete (>196 kb) MPXV genomes (*n* = 2108, as of 21 August 2025) were downloaded from GISAID EpiPox [30], excluding low-coverage sequences (>5% Ns).

To investigate the phylogenetic evolution, a Maximum Likelihood (ML) phylogenetic tree was reconstructed using a dataset including our samples and the complete sequences available from the GISAID repository, for a total of 2148 records. Multiple alignment was performed with MAFFT v7.523 [47], and ML phylogeny was inferred using IQ-TREE v2.2.2.7 [48] and visualized in iTOL v6.9.1 [49]. To investigate STR similarities between samples, we performed a hierarchical clustering using the number of STRs belonging to each sample matrix (Euclidean distance, R packages ape v.5.8.1, dendextend v.1.19.1, and ggtree v.3.16.3) [50,51,52].

#### 4.2.4. Protein Translation and In Silico Structural Prediction

Genomic sequences containing STRs located within protein-coding regions were translated into amino acid sequences using the EMBOSS v.6.6.0.0 [53] executed from the command line. Translation was performed according to the standard genetic code, preserving the annotated reading frame and strand orientation. The resulting protein was compared to its corresponding wild-type reference, derived from UniProt [54] annotations of the same gene. When sequence alterations were detected relative to the reference, the affected open reading frames were subjected to three-dimensional structure prediction using AlphaFold2 [55] (ab initio modeling). In particular, the OPG153 gene (GeneID: 72551547) and its Δ4D variant were modeled independently. Structural visualization and overlay were carried out using Chimera tool v.1.19 [56].

## Figures and Tables

**Table 1:** Comparison of STRs identified in this study with those reported by Monzòn et al. (2024) [24]. The table summarizes the genomic location, repeat unit, repeat counts, and functional annotation for each STR identified in MPXV genomes from this study. The second column indicates the corresponding STRs described by Monzòn et al. (2024) [24]. Entries marked with an asterisk (*) denote STRs that overlap with a slightly different sequence composition than those reported in Palacios et al. NA indicates not available information.

Name	Correspondence with Monzòn et al. [24]	Start	End	Pattern	# Max Rep	Str length (bp)	# Samples Sharing the STR	Nearest Gene	Relative Position to the Gene	Distance in bp	Functional Insight
**STR-I**	LCR10	595	611	[T]	14	14	40/40	OPG001	Upstream	−224	Chemokine-binding protein
**STR-II**	LCR1 *	4677	4801	[TAACTAACTTATGACT]	9	144	40/40	OPG015	Downstream	37	Ankyrin domains and immune evasion (TNF mimic)
**STR-III**	LCR12	28,527	28,537	[A]	9	9	40/40	OPG044	Intragene	NA	Bcl-2-like protein
**STR-IV**	LCR13	76,082	76,090	[T]	9	9	40/40	OPG097	Intragene	NA	Unknown
**STR-V**	LCR14	80,844	80,825	[T]	9	9	40/40	OPG104	Intragene	NA	Unknown
**STR-VI**	LCR5	133,074	133,098	[T]	25	25	40/40	OPG151	Upstream	−657	Unknown
**STR-VII**	LCR7	136,498	136,555	[ATC]n + TATGAT + [ATC]n	19	57	40/40	OPG153	Intragene	NA	Key attachment/egress protein
**STR-VIII**	LCR15	140,092	140,131	[ATAACAATT]	4.4	39	40/40	OPG159	Intragene	NA	Unknown
**STR-IX**	LCR8	146,835	146,920	[ATATTTT]n + [ATTTT]n	10	64	40/40	OPG170	Upstream	−57	Unknown
**STR-X**	LCR9 *	150,544	150,621	[GATATGATGGATATGAT]n + [GGATATGAT]n	5	62	40/40	OPG176	Intragene	NA	Bcl-2-like protein
**STR-XI**	LCR16	152,661	152,566	[A]	7	9	40/40	OPG180	Upstream	−31	Unknown
**STR-XII**	LCR17	1631,69	163,207	[TAAC]	6	24	40/40	OPG188	Upstream	−63	Unknown
**STR-XIII**	LCR18	166,070	166,109	[AATAATT]	3.7	39	40/40	OPG190	Intragene	NA	IFNα/β binding protein homolog
**STR-XIV**	LCR19 *	169,709	169,761	[CAGATA]	8.8	52	40/40	OPG197	Intragene	NA	Unknown
**STR-XV**	LCR2 *	173,252	173,295	[AT]	22	43	40/40	OPG200	Upstream	−654	Unknown
**STR-XVI**	LCR21	174,506	174,538	[GATGAA]	4.8	32	40/40	OPG204	Intragene	NA	Interferon decoy receptor; immune modulation
**STR-XVII**	LCR3 *	179,055	179,245	[ATATACATT]	16	144	40/40	OPG208	Downstream	34	Serine protease inhibitor (anti-apoptotic, potential virulence)
**STR-XVIII**	LCR4	192,382	192,534	[AGTCATAAGTTAGTTA]	9	144	40/40	OPG015	Upstream	−16	Ankyrin domains and immune evasion (TNF mimic)
**STR-XIX**	LCR11 *	196,608	196,624	[A]	14	14	38/40	OPG001	Downstream	233	Chemokine-binding protein

**Table 2:** Patients’ general information. The table reports the details of each sample matrix, the collection date, the travel history, and the qPCR Ct. NA indicates not available information.

P.	Matrix	Timepoint	Sample_Id	Collection_Date	Travel_History	qPCR
**P1**	Skin lesion swab	I	P1_SL_I	25 May2022	Mallorca	21.00
**P1**	Skin lesion swab	II	P1_SL_1_II	03 June 2022	Mallorca	21.51
**P1**	Skin lesion swab	III	P1_SL_2_III	07 June 2022	Mallorca	21.00
**P2**	Skin lesion swab (genital)	I	P2_SLG_I	18 July 2022	Spain	18.96
**P2**	urine	I	P2_U_I	18 July 2022	Spain	25.91
**P2**	Rectal swab	I	P2_R_I	18 July 2022	Spain	18.52
**P2**	Skin lesion swab (genital)	I	P2_SLG_2_I	18 July 2022	Spain	17.07
**P2**	Skin lesion swab (genital)	II	P2_SLG_II	21 July 2022	Spain	20.96
**P2**	Rectal swab	II	P2_R_II	21 July 2022	Spain	20.63
**P3**	Skin lesion swab	I	P3_SL_I	18 October 2022	None	14.70
**P3**	Pharyngeal swab	I	P3_P_I	18 October 2022	None	16.01
**P3**	Saliva	I	P3_S_I	18 October 2022	None	20.06
**P3**	Skin lesion swab (head)	I	P3_SL_2_I	18 October2022	None	18.99
**P4**	Skin lesion swab (abdomen)	I	P4_SL_I	02 November 2022	Bosnia-Herzegovina	16.70
**P4**	Skin lesion swab (hand)	I	P4_SL_2_I	02 November 2022	Bosnia-Herzegovina	14.40
**P4**	Skin lesion swab (head)	I	P4_SL_3_I	02 November 2022	Bosnia-Herzegovina	14.00
**P4**	Pharyngeal swab	I	P4_P_I	02 November 2022	Bosnia-Herzegovina	23.10
**P4**	Urine	I	P4_U_I	02 November 2022	Bosnia-Herzegovina	25.80
**P4**	Skin lesion swab (leg)	II	P4_SL_II	02 November 2022	Bosnia-Herzegovina	25.30
**P4**	Urine	II	P4_U_II	02 November 2022	Bosnia-Herzegovina	25.76
**P5**	Skin lesion swab	I	P5_SL_I	23 June 2022	Spain	22.19
**P5**	Rectal swab	I	P5_R_I	23 June 2022	Spain	18.78
**P6**	Skin lesion swab	I	P6_SL_I	21 July 2022	None	26.65
**P7**	Skin lesion swab (genital)	I	P7_SLG_I	01 July 2022	None	21.02
**P8**	Skin lesion swab (genital)	I	P8_SLG_I	15 July 2022	None	19.83
**P8**	Skin lesion swab (genital)	I	P8_SLG_2_I	15 July 2022	None	15.05
**P8**	Skin lesion swab (genital)	II	P8_SLG_II	21 July 2022	None	19.07
**P8**	Urine	II	P8_SLG_2_II	21 July 2022	None	25.10
**P9**	Skin lesion swab	I	P9_SL_I	01 July 2022	USA	26.67
**P9**	Skin lesion swab	II	P9_SL_2_II	01 July 2022	USA	22.74
**P10**	Skin lesion swab	I	P10_SL_I	26 July2022	None	24.05
**P10**	Skin lesion swab	II	P10_SL_II	29 July 2022	None	19.05
**P11**	Skin lesion swab (abdomen)	I	P11_SL_I	05 August 2022	None	20.17
**P11**	Skin lesion swab (arm)	I	P11_SL_2_I	05 August 2022	None	19.32
**P12**	Skin lesion swab (head)	I	P12_SL_I	18 August 2022	None	18.06
**P12**	Rectal swab	I	P12_R_I	18 August 2022	None	25.25
**P13**	Skin lesion swab	I	P13_SL_I	19September 2022	None	21.31
**P15**	Pharyngeal swab	I	P15_P_I	08 July 2022	None	27.03
**P16**	Skin lesion swab	I	P16_SL_I	NA	NA	NA
**P19**	Skin lesion swab	I	P19_SL_I	NA	NA	NA

## Data Availability

The data presented in this study are openly available in [GISAID] at [https://www.epicov.org/epi3/frontend#29d92e, accessed on 28 January 2026]. The correspondence between the experimental ID (reported in this manuscript) and the GISAID IDs (EPI_IDs) is provided in Supplementary Materials, Table S3. The raw data supporting the conclusions of this article will be made available by the authors on request.

## Supplementary Materials

The following supporting information can be downloaded at: https://www.mdpi.com/article/10.3390/ijms27031371/s1.
