# Supplementary material for: Insights into Genomic Dynamics and Plasticity in the Monkeypox Virus from the 2022 Outbreak
Source: Int J Mol Sci. 2026 Jan 29;27(3):1371. doi: 10.3390/ijms27031371 (PMC12898169; doi:10.3390/ijms27031371)
Supplement: Supplementary file 1 [file ijms-27-01371-s001.zip › Supplementary_materials.pdf]

Supplementary Figure S1. Multiple sequence alignment of amino acid sequences corresponding to STR-containing regions. Red boxes indicate amino acid residues encoded by or directly affected by STR loci. OPG176 and OPG190 d are not shown, as their associated repeat regions are located outside the protein-coding sequence

OPG044

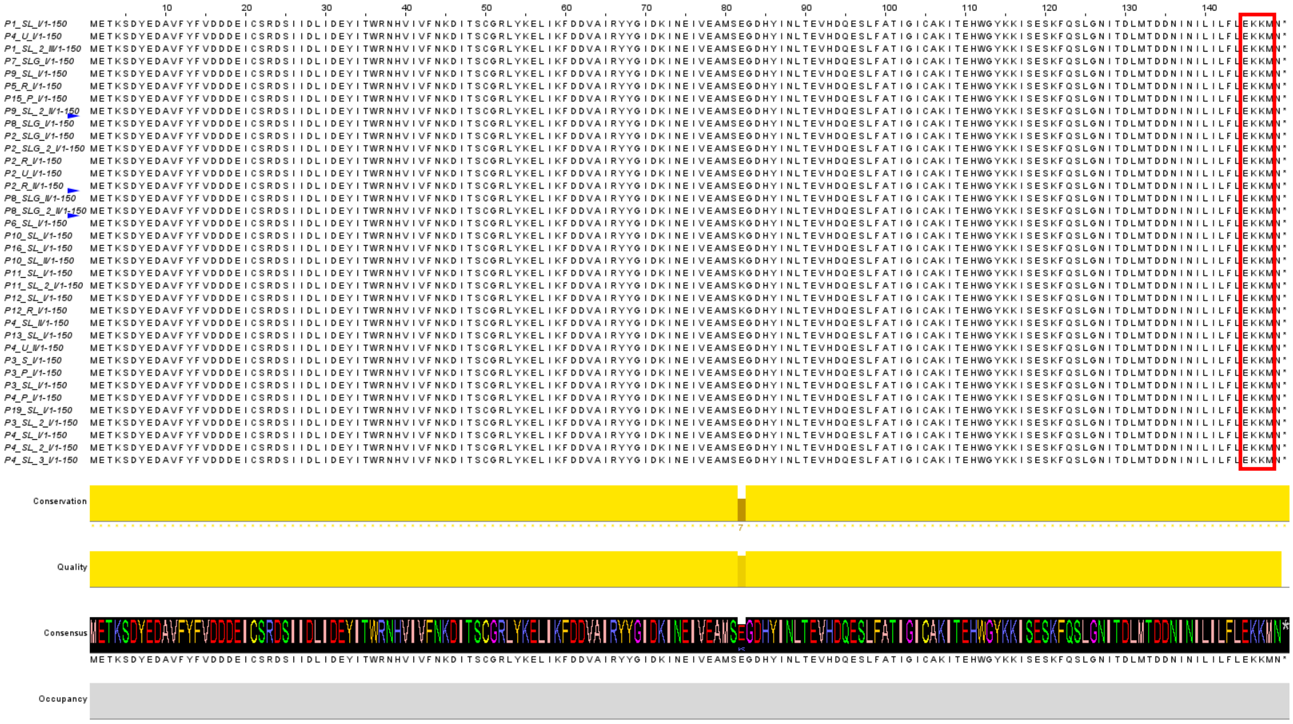

OPG097

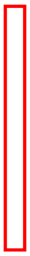

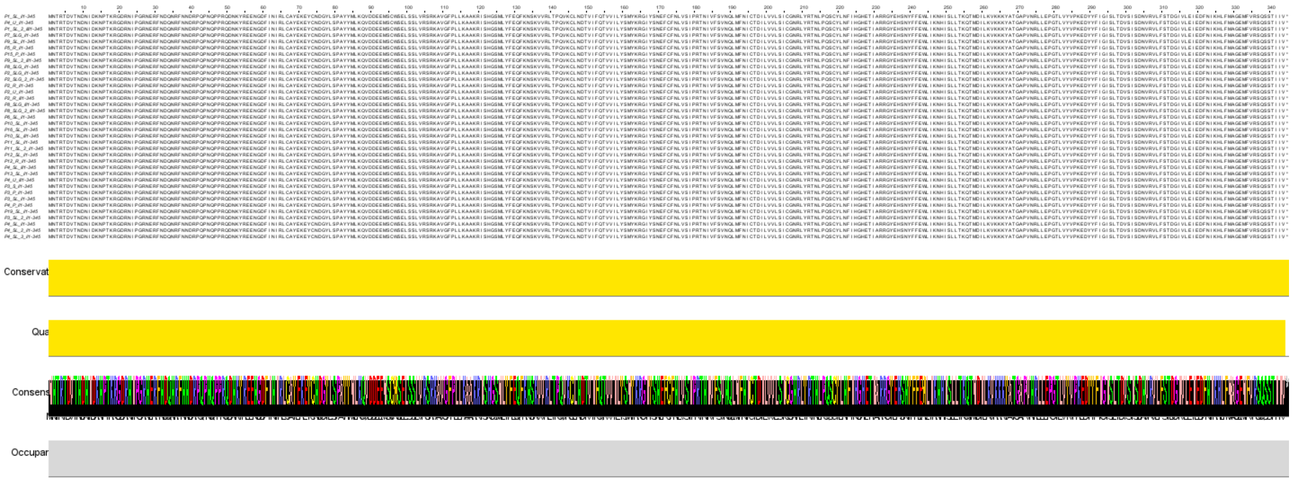

OPG104

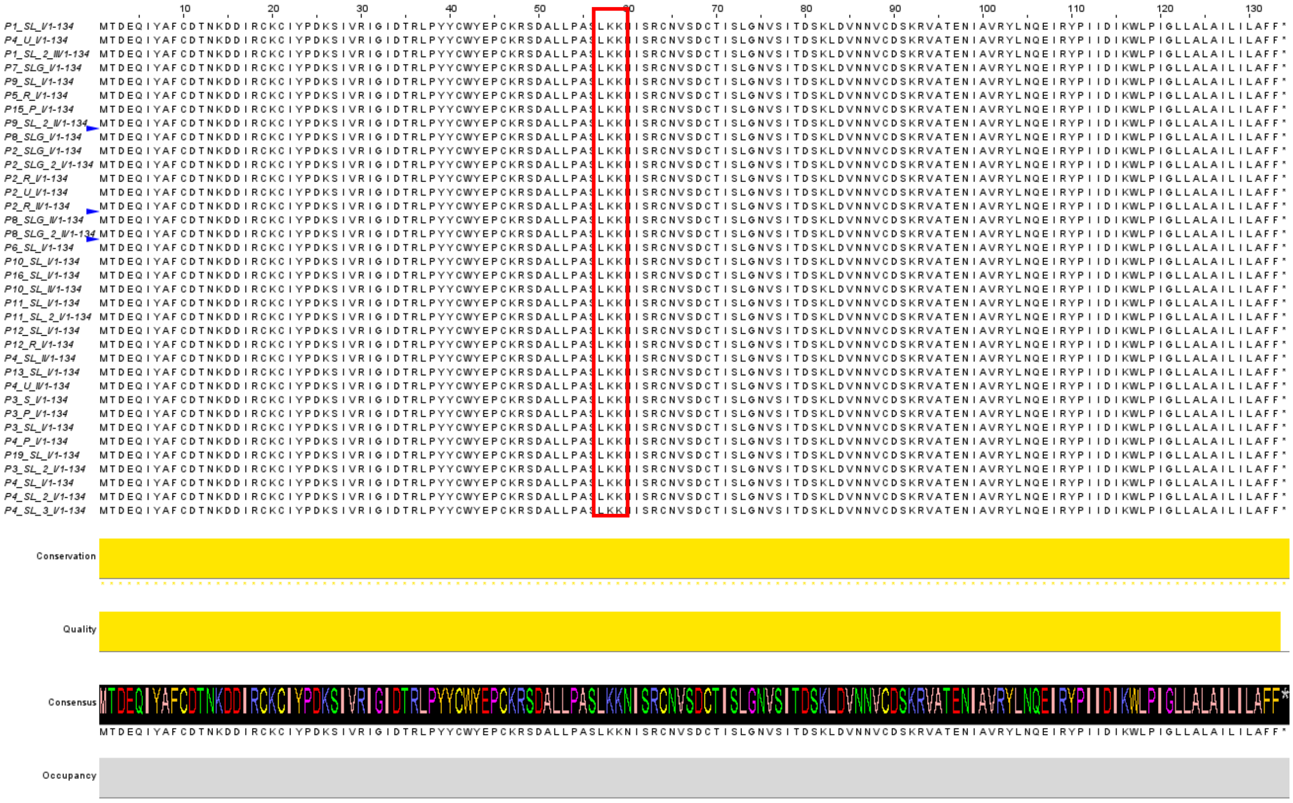
10 20 30 40 50 60 70 80 90 100 110 120 130
P1_SL_I/1-134
P4_U_I/1-134
P1_SL_2_III/1-134
P7_SLG_I/1-134
P9_SL_I/1-134
P5_R_I/1-134
P15_P_I/1-134
P9_SL_2_II/1-134
P8_SLG_I/1-134
P2_SLG_I/1-134
P2_SLG_2_I/1-134
P2_R_I/1-134
P2_U_I/1-134
P2_R_II/1-134
P8_SLG_II/1-134
P8_SLG_2_II/1-134
P6_SL_I/1-134
P10_SL_I/1-134
P16_SL_I/1-134
P10_SL_II/1-134
P11_SL_I/1-134
P11_SL_2_I/1-134
P12_SL_I/1-134
P12_R_I/1-134
P4_SL_II/1-134
P13_SL_I/1-134
P4_U_II/1-134
P3_S_I/1-134
P3_P_I/1-134
P3_SL_I/1-134
P4_P_I/1-134
P19_SL_I/1-134
P3_SL_2_I/1-134
P4_SL_I/1-134
P4_SL_2_I/1-134
P4_SL_3_I/1-134
Conservation
Quality
Consensus
MTDEQIYAFCDTNKDDIRCKCIYPDKSIVRIGIDTRLPYYCWYEPCKRSDALLPASLKKNISRCNVSDCTISLGNVSITDSKLDVNNVCDSKRVATENIAVRYLNQEIRYPIIDIKWLPIGLLALAILILAFF*
Occupancy

OPG159

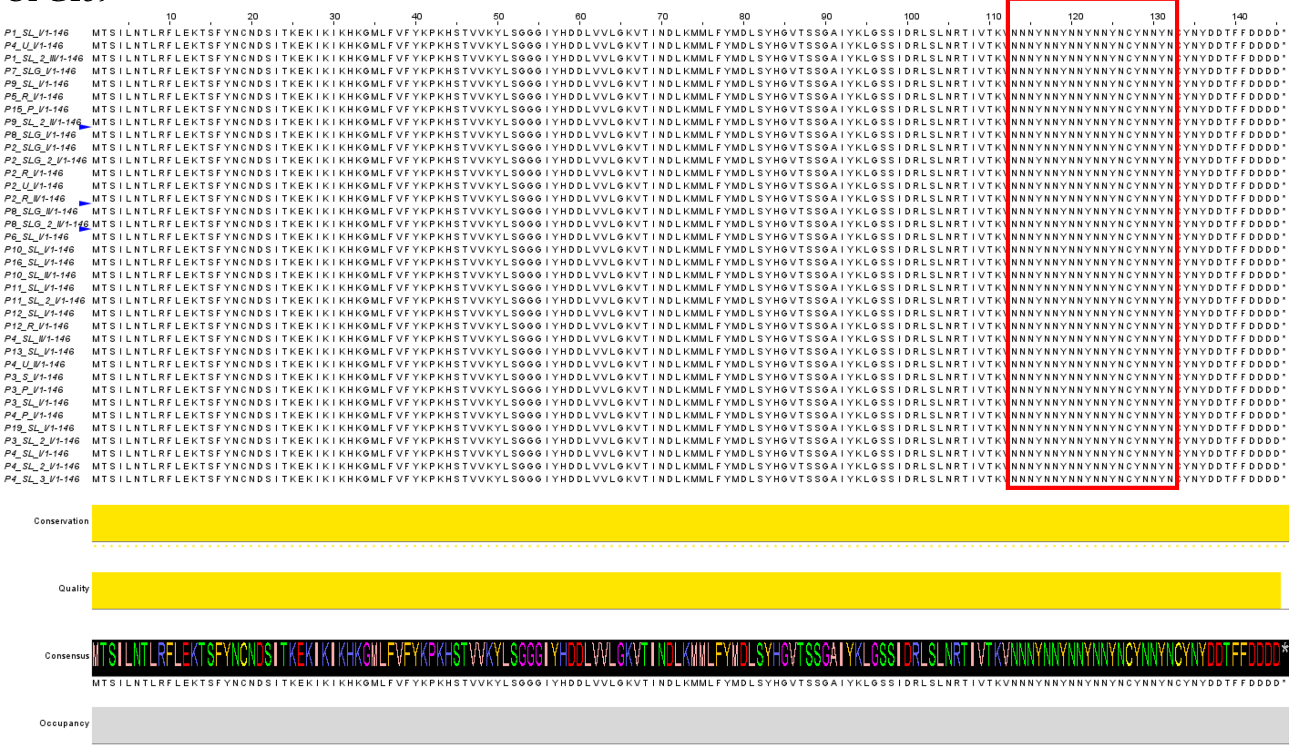

OPG197

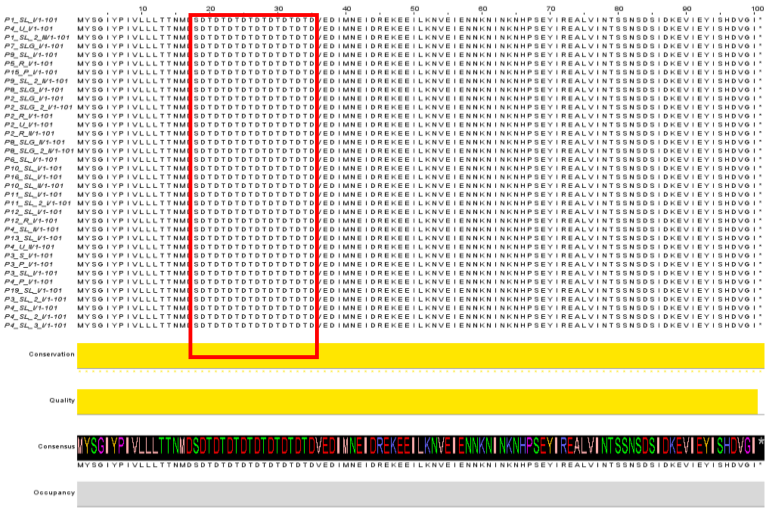

OPG204

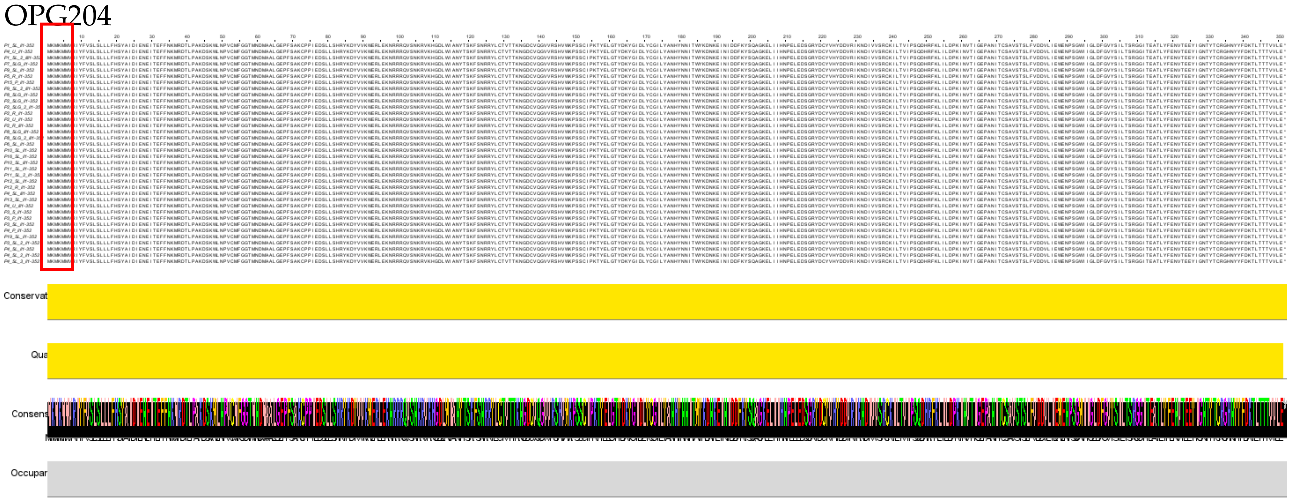

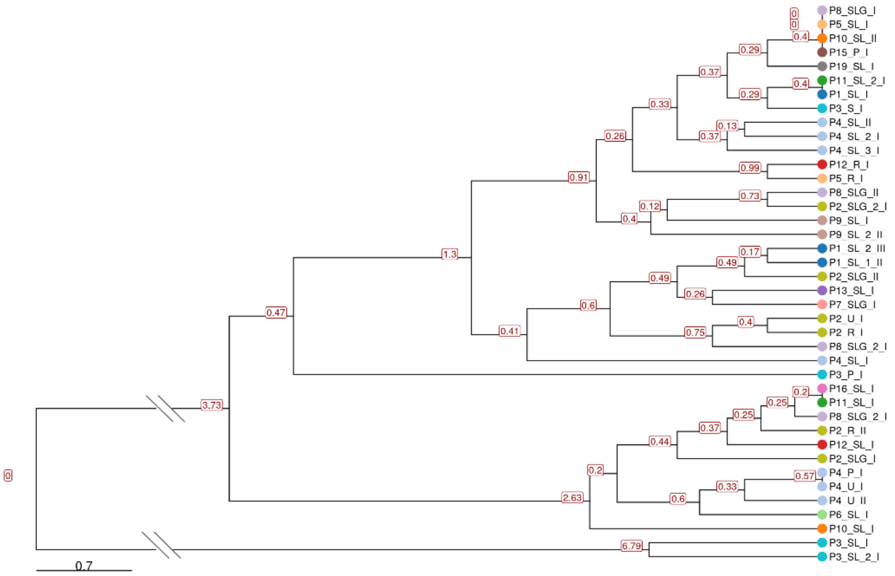

Supplementary figure_S2. Hierarchical clustering of STR profiles. The dendrogram is based on Euclidean distances, which reflect the dissimilarity between STR repeat copy numbers. Shorter distances indicate higher similarity in STR patterns between samples, while longer distances reflect greater divergence. The clustering is derived from repeat copy numbers and does not represent nucleotide sequence relationships.
